# Survival Prediction for Non–Asphyxia-Related Hypothermic Cardiac Arrest Patients After Extracorporeal Rewarming: Development of the HELP Score

**DOI:** 10.1097/MAT.0000000000002456

**Published:** 2025-05-14

**Authors:** Paweł Podsiadło, Konrad Mendrala, Les Gordon, Mathieu Pasquier, Peter Paal, Hubert Hymczak, Anna Witt-Majchrzak, Ewelina Nowak, Tomasz Czarnik, Tomasz Darocha

**Affiliations:** From the * Department of Emergency Medicine, Jan Kochanowski University, Kielce, Poland; † Department of Anaesthesiology and Intensive Care, Medical University of Silesia, Katowice, Poland; ‡ Department of Anaesthesia, University Hospitals of Morecambe Bay Trust, Lancaster, United Kingdom; § Emergency Department, Lausanne University Hospital, and University of Lausanne, Lausanne, Switzerland; ¶ Department of Anaesthesiology and Intensive Care Medicine, Hospitallers Brothers Hospital, Paracelsus Medical University, Salzburg, Austria; ∥ Department of Anaesthesiology and Intensive Care, Andrzej Frycz Modrzewski Krakow University, Krakow, Poland; # Department of Cardiac Surgery, Provincial Specialist Hospital, Olsztyn, Poland; ** Faculty of Health Sciences, Jan Kochanowski University, Kielce, Poland; †† Department of Anaesthesiology and Intensive Care, Institute of Medical Sciences, University of Opole, Opole, Poland.

**Keywords:** accidental hypothermia, cardiac arrest, extracorporeal life support, patient outcome assessment, resuscitation

## Abstract

The aim of this study was to develop a scoring tool to estimate the probability of survival following extracorporeal rewarming in patients suffering hypothermic cardiac arrest. This is a multicenter retrospective study based on registry data. We included adult patients with hypothermic cardiac arrest not associated with asphyxia, with a core temperature of ≤28°C, who underwent extracorporeal rewarming. A multivariable logistic regression model was developed to serve as the predictive tool. Internal validation with bootstrap resampling was performed to adjust model parameters and reduce model optimism. Our study population included 141 patients. The survival rate was 46% (65/141). A total of 88% of the survivors (57/65) had a favorable neurological outcome (Cerebral Performance Category 1–2). The predictive model includes four variables. Outdoor occurrence of hypothermia and a higher hemoglobin level raise survival odds while higher concentrations of potassium and lactate reduce survival odds. The area under the receiver operating characteristic (ROC) curve was 0.812 and *p* value of the Hosmer-Lemeshow test was 0.8. We developed a prognostic model to estimate the probability of survival in adult patients with non–asphyxia-related hypothermic cardiac arrest. This model may aid in identifying candidates suitable for extracorporeal rewarming, though it should not be used as the sole deciding factor.

Compared with normothermic cardiac arrest, hypothermic cardiac arrest (HCA) is associated with a higher survival rate with favorable neurological outcome.^1^ Rewarming with Extracorporeal Life Support (ECLS) is deemed to be the optimal treatment for HCA.^2,3^ However, identifying patients suitable for this method is challenging. To date, four tools have been developed to predict the outcome in hypothermic patients and support clinicians in decision-making: Hypothermia Outcome Score, 5A, Hypothermia Outcome Prediction after ECLS (HOPE), ICE.^4–7^

Victims of accidental hypothermia are a diverse population ranging from young healthy mountaineers to elderly individuals with multiple comorbidities who develop secondary hypothermia indoors. In an urban environment, homeless people and victims of alcohol and recreational drug abuse are a substantial part of this population.^8–10^ Since people are living longer in developed countries, older people with frailty are seen more frequently. Frailty is a multifactorial syndrome characterized by reduced physiological reserve and an increased vulnerability to stressors.^11,12^ However, prediction tools developed from a broader range of patient populations including young, asphyxiated victims of drowning or avalanche burial, are unlikely to be reliable for estimating survival chances in patients from an urban environment, particularly the old and frail.

Given the limited availability of ECLS, improved outcome prediction could lead to a better use of scarce resources and optimize patient selection. An accurate prediction would also help centers who get little experience with rewarming severely hypothermic patients to feel on safe ground when justifying a rewarming attempt. As with normothermic extracorporeal resuscitation, the decision to initiate ECLS in hypothermic patients should consider the likelihood of a favorable outcome.

The aim of this study was to develop a scoring tool for prediction of survival in patients suffering non–asphyxia-related HCA rewarmed with ECLS.

## Methods

### Study Design

We conducted a nationwide multicenter retrospective study using patient data derived from the HypothErmia Life Support in Poland (HELP) Registry that collects the data of consecutive patient series from collaborating hospitals in Poland. The development and internal validation of the predictive model adhered to the Transparent Reporting of a multivariable prediction model for Individual Prognosis Or Diagnosis (TRIPOD) Statement requirements.^13^ The TRIPOD checklist is provided in Supplementary File 1, Supplemental Digital Content, https://links.lww.com/ASAIO/B498. This study was approved by Jan Kochanowski University Bioethical Board, Kielce, Poland, Consent Number 31/2024.

### Patient Selection Criteria

We included adult (≥18 years old) victims of accidental hypothermia with a core temperature of ≤28°C on admission to hospital, who underwent ECLS rewarming (venoarterial extracorporeal membrane oxygenation or cardiopulmonary by-pass) due to HCA between January 2014 and April 2024.

Exclusion criteria were patients with cardiac arrest from causes other than hypothermia, traumatic brain injury or recent brain stroke, known metastatic cancer, cooling with pre-arrest asphyxia (*e.g*., avalanche burial with cardiac arrest at extrication, drowning, *etc*.), and missing information on the outcome.

### Outcome

Survival to hospital discharge was the primary outcome as a binary variable (in-hospital death or survival to hospital discharge). The secondary outcome was neurological status at discharge, assessed using the Cerebral Performance Category (CPC) score.^14^ A CPC of 1 or 2 was considered as a favorable neurological outcome.

### Collected Data

Age; sex; core temperature (Tc) on admission to the ECLS center (measured in emergency department or in operating room, in the esophagus, rectum, bladder, or on tympanic membrane with a thermistor); circumstances of hypothermia development (indoors or outdoors); cardiac arrest rhythm; circumstances of cardiac arrest occurrence (witnessed or unwitnessed); duration of cardiopulmonary resuscitation (CPR) before ECLS initiation; arterial blood pH on admission to the ECLS center (not-corrected for patient body temperature); initial hemoglobin, serum potassium and serum lactate concentrations measured from arterial blood, and patient outcome.

### Data Analysis

The study population was divided into two groups: survivors and nonsurvivors. The groups were compared by analyzing collected variables regarding their association with the outcome. Heart rhythm was expressed as three dichotomous variables: asystole *versus* others, pulseless electrical activity (PEA) *versus* others, and ventricular fibrillation (VF) *versus* others.

Our dataset had 2.4% missing values. They were considered missing-at-random and imputed using the Multiple Imputation module of the SPSS statistical package. We generated 100 datasets with imputed data performing 100 iterations of the algorithm for each dataset.^15^

Univariable analysis was conducted using logistic regression, presenting odds ratios with 95% confidence intervals and *p* values. One variable, namely blood pH, was log-transformed due to its nonlinear relationship with the outcome.

We used a two-way strategy of multivariable model building: automated backward elimination and user-controlled selection.^16,17^ Automated backward elimination was based on Wald statistics. Candidate predictors were selected considering their association with the outcome in univariable analysis at a *p* value threshold of ≤0.25 (Table 1). Interaction terms for independent variables, automatically suggested by the statistical software, were also considered as candidate predictors. For manual model building, we used the Stepwise Model Builder (Statistica package) which allows user-controlled selection of any variables, and their introduction into the model, including those that were not statistically significant but were considered clinically important (*e.g*., sex, asystole, and PEA). The developed models were compared based on their discrimination and calibration, using the area under the receiver operating characteristic curve (AUROC) and the Hosmer-Lemeshow test. Since the number of survivors in our study population was 65, the models including more than six independent variables were not considered acceptable due to potential risk of overfitting. The model with the highest AUROC and the best calibration assessed with the Hosmer-Lemeshow test was presumed to be optimal.

**Table 1.** Characteristics of Study Population and Univariable Analysis of Potential Predictors of Survival

	Missing Data	Overall (n = 141)	Survivors (n = 65)	Nonsurvivors (n = 76)	OR (95% CI)	*p*
Age (years)	2 (1%)	55 [46–63]	54 [41–60]	57 [48–68]	0.96 (0.94–0.99)	**0.01**
Tc (°C)	-	24 [22–25]	23.7 [22–24.2]	24 [22.2–25.9]	0.81 (0.69–0.95)	**0.01**
CPR duration (min)	7 (5%)	134 [80–190]	131.5 [63–158]	142 [85–220]	0.995 (0.99–1)	**0.02**
Blood pH	3 (2%)	7.032 [6.911–7.138]	7.046 [6.939–7.141]	7.009 [6.830–7.130]	4.2 (0.69–25.7)	0.1
Hemoglobin (g/dl)	6 (4%)	11.1 [9.6–12.9]	12 [10–14]	10.3 [8.8–11.8]	1.29 (1.11–1.49)	**<0.001**
Serum potassium (mmol/L)	2 (1%)	3.9 [3.1–5]	3.7 [3.0–4.5]	4.2 [3.4–5.6]	0.74 (0.57–0.96)	**0.02**
Serum lactate (mmol/L)	7 (5%)	9.8 [7–13.2]	8 [6.3–11.1]	12 [8–14.7]	0.85 (0.77–0.93)	**<0.001**
Hypothermia developed outdoors	8 (6%)	103 (77%)	55 (89%)	48 (68%)	3.77 (1.49–9.55)	**0.005**
Sex (male)	-	122 (86%)	56 (86%)	66 (87%)	0.94 (0.36–2.48)	0.9
Witnessed CA	3 (2%)	98 (71%)	48 (76%)	50 (67%)	1.6 (0.75–3.4)	0.2
Asystole	2 (1%)	35 (25%)	14 (21%)	21 (28%)	0.69 (0.32–1.51)	0.4
PEA	2 (1%)	10 (7%)	3 (5%)	7 (9%)	0.46 (0.12–1.87)	0.3
VF	2 (1%)	95 (68%)	48 (74%)	47 (63%)	1.62 (0.78–3.36)	0.2

Data are presented as median (interquartile range) and numbers (percentages).

Statistically significant values are in bold. CA, cardiac arrest; CI, confidence interval; CPR, cardiopulmonary resuscitation; OR, odds ratio; PEA, pulseless electrical activity; Tc, core body temperature; VF, ventricular fibrillation.

Both selection procedures resulted in the same optimal model. This model, including an entire predictor selection procedure, was applied to each imputed dataset resulting in 100 sets of predictor parameters, which were averaged using SPSS software.^17^ These averaged estimates of predictors constituted our predictive model.

We performed an internal validation with bootstrap method, testing our model on 100 resamples with replacement for each imputed dataset, resulting in a total of 10,000 resamples. The average estimates of predictors from the bootstrap resamples were used to reassess model performance and determine the shrinkage factor. This factor was calculated as the average slope of the linear predictor fitted to the original data, ensuring adjusted coefficients to improve model calibration and reduce overfitting.^18^ The difference between the mean AUROC from bootstrap resamples and the mean AUROC from model reassessment on imputed datasets was the measure of the optimism excess of our model. The regression coefficients for the final model were corrected using the shrinkage factor, as described by Steyerberg.^18^ We used IBM SPSS Statistics, v29.0.1.0 (IBM, Armonk, NY) and Statistica package version 13.0 (Tibco Software Inc).

## Results

### Study Population Characteristics

**Figure 1.**
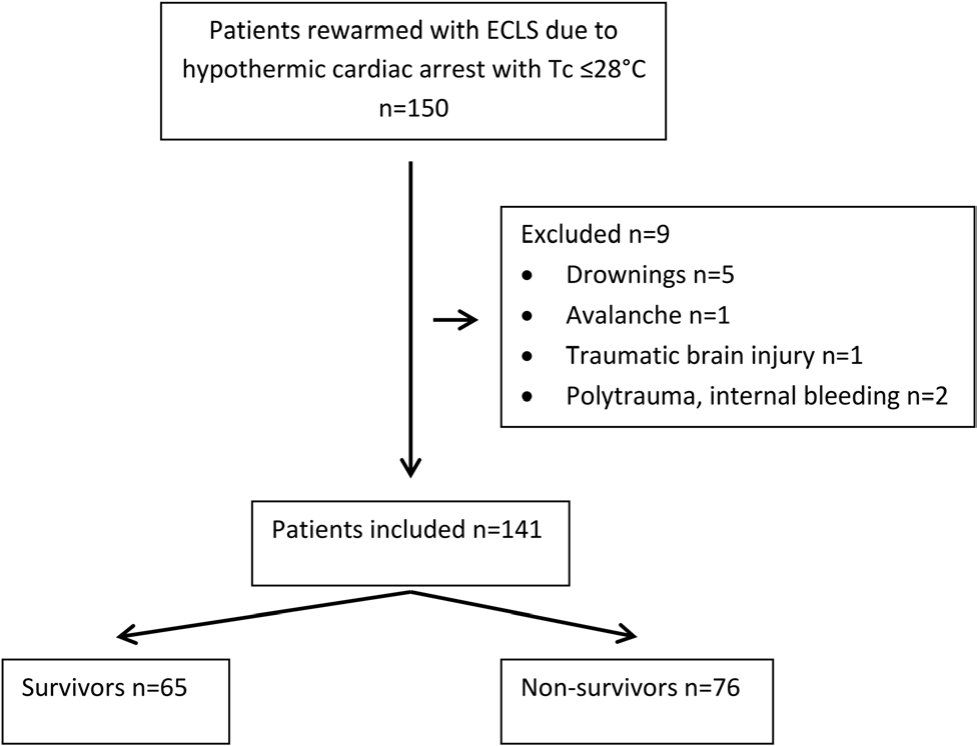
Flowchart of the study population. ECLS, extracorporeal life support; Tc, core body temperature.

A total of 150 patients were screened, of whom 141 were included in the study (Figure 1). Sixty-five of the 141 patients (46%) survived hospital discharge. Fifty-seven of the 65 (88%) survivors had a CPC of 1 or 2 at hospital discharge. The remaining eight survivors (12%) had a poor neurological outcome, with a CPC of 3 or 4.

### Predictors of Survival

Univariable association between collected variables and the outcome are presented in Table 1.

The final model consisted of four predictors: circumstances of hypothermia development (indoors or outdoors), hemoglobin, potassium, and lactate concentrations. The details of multivariable analysis are summarized in Table 2. The AUROC of the original model was 0.812 (AUC error of 0.038), demonstrating excellent discriminative ability.^19^ The Hosmer-Lemeshow test *p* value was 0.8, showing good calibration. After internal validation and reduction of optimism, the corrected AUROC was 0.801.

**Table 2.** Variables Included in the Optimal Prediction Model

Variable	Estimate	OR (95% CI)	*p*
Hemoglobin (g/dl)	0.346	1.41 (1.17–1.71)	<0.001
Serum potassium (mmol/L)	−0.386	0.68 (0.48–0.96)	0.03
Serum lactate (mmol/L)	−0.207	0.81 (0.73–0.91)	<0.001
Hypothermia developed outdoors	1.67	5.31 (1.61–17.58)	0.006

CI, confidence interval; OR, odds ratio.

The final equation for the HELP score (Hemoglobin, Exposure, Lactate, Potassium), incorporating predictor estimates corrected after internal validation and enabling the calculation of survival probability, is as follows:


$$\begin{array}{l} \begin{array}{l} Outcomevariable=-1.626+1.456\\ \times \left(outdooroccurrenceofhypothermia\right)\\ +0.317\times \left(hemoglobin\right)-0.354\\ \times \left(potassium\right)-0.183\times (lactate) \end{array} \end{array}$$

The calculator transformed into the formula for spreadsheet returning probability of survival is as follows:


$$\begin{array}{l} \begin{array}{l} =EXP\left(\begin{array}{l} -1,626+1,456\times outdoors+0.317\\ \times hemoglobin-0.354\times potassium-0.183\times lactate \end{array}\right)/\\ \left(1+EXP\left(\begin{array}{l} -1,626+1,456\times outdoors+0.317\\ \times hemoglobin-0.354\times potassium-0,183\times lactate \end{array}\right)\right) \end{array}, \end{array}$$

where “outdoors” denotes the site of hypothermia development: outdoors = 1, indoors = 0; “hemoglobin” denotes hemoglobin concentration (g/dl); “potassium” denotes potassium concentration (mmol/L); and “lactate” denotes lactate concentration (mmol/L).

**Figure 2.**
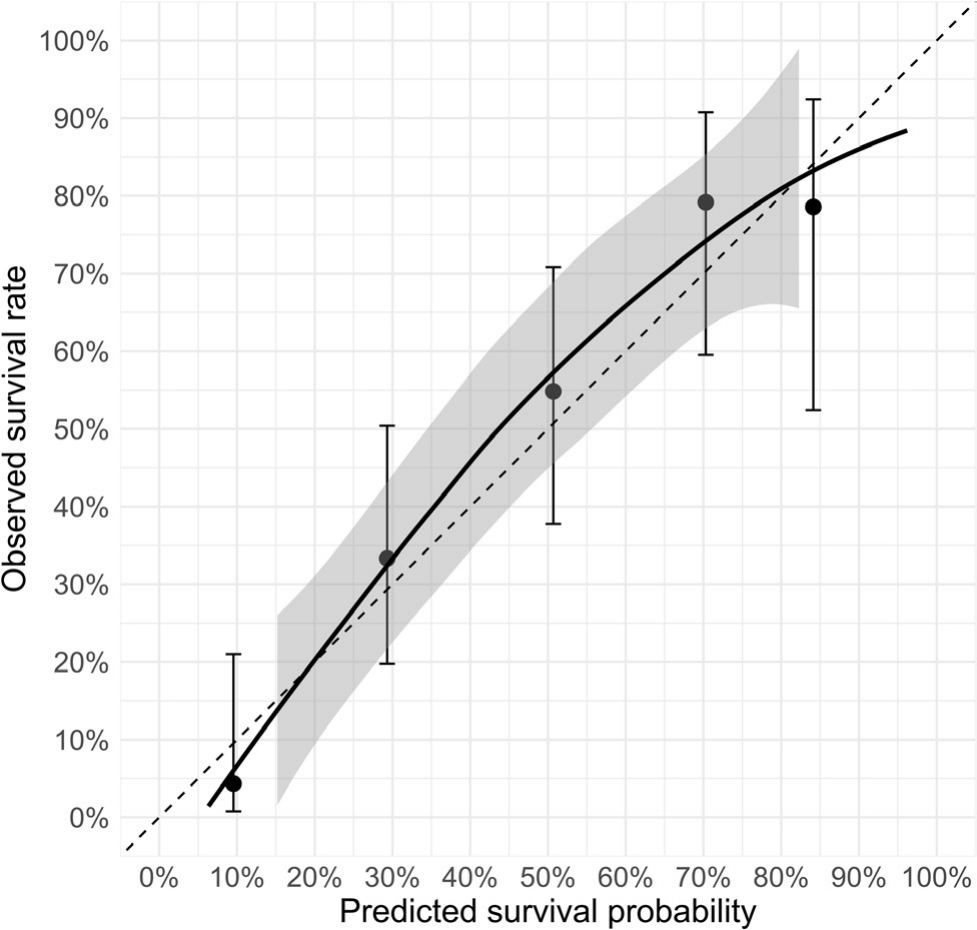
Calibration plot comparing predicted and observed survival probabilities. Points represent mean observed survival with 95% confidence intervals. The dashed line indicates perfect calibration, the black curve shows LOESS calibration with a 95% confidence interval gray band. Calibration intercept = 0.12, calibration slope = 1.116. LOESS, locally estimated scatterplot smoothing.

The actual survival rates of our patients within quantiles of predicted probability of survival are presented in Figure 2.

## Discussion

Using multivariable logistic regression, we have developed a scoring tool that can potentially help clinicians in decision-making for ECLS. Four key survival predictors were identified: hypothermia occurring outdoors is associated with a 5.3-fold increase in survival odds, and each 1 g/dl increase in hemoglobin raises survival odds by 1.4-fold. Conversely, each 1 mmol/L increase in potassium and lactate reduce survival odds by 1.5- and 1.2-fold, respectively.

Two of the aforementioned predictive tools pertaining to ECLS treatment were developed from younger patient populations. In the HOPE-score and ICE-score studies, the median age was 35 and 36 years, respectively.^5,6^ They also included a substantial proportion of asphyxia victims (51% and 43%, respectively). Accidental hypothermia victims in an urban environment are usually substantially older, and this has been shown in several studies. Roeggla *et al*. (Vienna) reported a median age of 55 years, Mégarbane *et al*. (Paris) 56.5 years, Vassal *et al*. (Paris) 61.7 years, Okada *et al*. (Japan) 79 years.^7,20–22^ The median age in our study population was 55 years. In addition, the nature of urban/rural environments means that an asphyxia-related mechanism of cooling is rare. Therefore, other clinical and biochemical parameters may have a more important role in affecting outcome.

High lactate concentration has already been proven to predict poor outcomes in several studies on HCA and normothermic out-of-hospital cardiac arrest.^6,23–26^ In our analysis, lactate concentration, reflecting the metabolic effect of tissue hypoperfusion due to prolonged cardiac arrest, proved to be a better survival predictor than the duration of cardiac arrest itself. The limited prognostic value of CPR duration in HCA has been shown by Shoji *et al*. and Darocha *et al*.^23,27^ It seems likely that an individual patient metabolic response to the prolonged hypoxia can better predict the outcome than the duration of hypoperfusion. Notably, the upper limit of Tc in our study population was 28°C which could diminish the predictive value of CPR duration, probably due to the protective effect of low temperature in hypoxia, as it is known that prolonged CPR does not preclude a good outcome in HCA.^28^

Low hemoglobin may reflect the general health status of patients and indicates their ability to recover after normothermic cardiac arrest.^29,30^ The relationship between hemoglobin concentration and outcome has also been observed in victims of accidental hypothermia. Danzl *et al*.^4,31^ in their two studies showed that hemoglobin concentrations are higher in survivors than in nonsurvivors (12.8 *vs*. 11.7 g/dl, *p* < 0.05 and 12.9 *vs*. 11.4 g/dl; *p* = 0.01). This association was confirmed in a study on accidental hypothermia in an urban environment conducted by Mégarbane *et al*. (13.3 *vs*. 12.3 g/dl; *p* = 0.08).^21^ Moreover, low hemoglobin level is significantly associated with frailty which may explain why it features in our score.^32^

Accidental hypothermia can develop in different circumstances. In outdoor settings, relatively healthy individuals can become hypothermic after being exposed to sufficient cold to overwhelm their metabolic heat production. This type of acute (sometimes called “exposure”) hypothermia commonly affects homeless or intoxicated people. Subchronic (sometimes called “urban”) hypothermia is usually seen in old and multimorbid patients with depleted physiological reserves, who often live in poor socioeconomic settings and become hypothermic in their homes.^33^ Indoor occurrence has already been shown to be significantly associated with an unfavorable outcome.^22,34,35^ In the study by Roeggla *et al*.,^20^ only indoor occurrence, blood urea, and platelet count proved to be independent predictors of mortality. The study by Mégarbane *et al*.^21^ similarly found that, for equivalent body temperatures, patients found indoors died more frequently than those found outdoors. Mendrala *et al*.^36^ suggest that indoor occurrence of hypothermia may be indicative of multimorbidity. Even if data was available on how comorbidities affect outcome, it is rarely possible to obtain a medical history from the patient before making a decision about ECLS so other sources of information are needed to guide decision-making.

Our score seems to accurately predict the probability of survival after HCA but is clearly not suitable for patients who became hypothermic in asphyxia-related conditions. Its additional advantage is that all biochemical parameters are readily available through point-of-care analysis. Further research is needed to define optimal target groups of patients for each of existing scoring tools.

### Limitations

This study suffers from several limitations. Its retrospective design is associated with nonuniform treatment across hospitals. Therefore, treatment results may differ between centers depending on personnel experience. Moreover, using registry data is associated with selection bias because patient reporting by hospitals is not obligatory so not all hospitals contributed data. Although we used an advanced imputation method, the lack of original data could affect our results and reduce transportability of a prediction model. Our analysis was based on a relatively small sample size compared with other studies on the same topic. This limited the number of predictors included in the multivariable model and may have hindered the detection of additional associations between variables. Therefore, external validation is needed to reassess the model’s performance. Given the limitations outlined earlier, caution is advised at the moment when using this prediction tool.

## Conclusions

We have developed a prognostic model to estimate survival probability in adult patients with non-asphyxia-related HCA, with a core temperature of ≤28°C, who are candidates for ECLS. Four survival predictors were identified: hypothermia occurring outdoors, a higher concentration of hemoglobin, and lower concentrations of serum potassium and lactate. This model may aid in identifying candidates for extracorporeal rewarming but should not be considered the sole deciding factor. External validation is needed to confirm its utility.

## Acknowledgments

The authors greatly thank the collaborators from the Hypothermia study group: Tomasz Jędrzejczak, Krzysztof Kępa, Dominik Drobiński, Barbara Barteczko-Grajek, Krzysztof Toczek, Wojciech Dąbrowski, Romuald Lango, Michał Pluta, Agnieszka Strzelecka, and Robert Sowiński.

## Supplementary Material
